# Maternal Risk Factors Associated with Preterm Births among Pregnant Women in Mogadishu, Somalia

**DOI:** 10.3390/children9101518

**Published:** 2022-10-04

**Authors:** Abdifetah Ibrahim Omar, Amina Dahir Mohamed, Mohamed Garad Farah, Ismail Abukar Mahad, Suban Abdullahi Mohamed, Abyan Hassan Dimbil, Nadifo Salad Mohamud, Fowziya Ahmed Abshir, Umayma Abdinasir Abdulkadir

**Affiliations:** 1 Faculty of Medicine and Health Sciences, Jamhuriya University of Science and Technology, Mogadishu 2526, Somalia; 2 Advance Medical Research Unit, Jamhuriya Research Center, Jamhuriya University of Science and Technology, Mogadishu 2526, Somalia

**Keywords:** maternal risk factors, FGM, preterm birth, Somalia

## Abstract

Background: Premature birth impacts millions of newborns annually. Sixty percent of the world’s preterm births occur in Sub-Saharan Africa and South Asia. Somalia’s premature birth rates and maternal risk factors are poorly studied; hence, this study aims to identify maternal risk factors related to premature births in Mogadishu, Somalia. Methods: This unmatched case-control study was conducted at four maternity hospitals in Mogadishu, Somalia. The cases were newborns with gestational ages of less than 37 weeks; controls were newborns with gestational ages of 37 to 42 weeks. All were live singletons. Cross-tabulation and binary logistic regression were used to analyze the data; a p-value of 0.05 was deemed statistically significant. Result: Of the total of 499 newborns, 70 were cases, and 429 were controls. Adequate prenatal care, maternal urine analysis, tetanus toxoid (TT) vaccination, hemoglobin (Hb) measurement, ultrasound monitoring, intake of iron + folic acid (IFA) supplement, blood pressure (BP) measurement during the current pregnancy, as well as partograph usage during labor all significantly decreased risk of having premature births. A prior history of preterm delivery and preeclampsia, obstetric complications, and female genital mutilation (FGM) significantly increased the risk of preterm births. Conclusion: The utilization of antenatal care services, use of a partograph, obstetric complications, and prior history of premature birth and preeclampsia had a substantial effect on preterm birth rates. This study identifies female genital mutilation (FGM) as a previously unidentified risk factor for preterm birth that needs additional investigation.

## 1. Introduction

Preterm birth (PTB) is a global health problem that affects millions of newborn babies each year [1,2]. PTB is defined as any newborn birth occurring prior to 37 weeks of pregnancy. It is classified by the World Health Organization into three categories: extremely premature, very premature, and moderate or late premature, at 28 weeks, between 28 and 32 weeks, and 32 to 37 weeks of pregnancy, respectively. Preterm delivery complications are estimated to be the leading cause of neonatal death [3].

Each year, over 130 million infants are born, almost 15 million of who are preterm, or more than one in every ten births globally. Over a million of these preterm infants die within the first few days of their lives. However, preterm birth rates vary substantially across the globe, with Northern Europe and Japan having the lowest rates while developing countries with low or middle incomes have the greatest rates [4,5]. Sub-Saharan African countries, in particular, account for over 60% of preterm births. Nigeria, Kenya, Malawi, and the Somali area of Ethiopia provide 23.7 percent, 18.3 percent, 16.3 percent, and 12.3 percent, respectively [1,4,5].

Several maternal-related risk factors have been associated with PTB, including lack of antenatal care (ANC) visits, obstetric complications, maternal age, nutritional status and maternal infection, gestational hypertension, a history of abortion, and low birth weight. All of these factors make pregnant women more susceptible to pregnancy-related disorders, which could contribute to PTB. Besides, urinary tract infection (UTI) and bacterial vaginitis are significantly associated with the risk of preterm delivery [6,7,8]. Furthermore, factors that contribute to preterm birth include preeclampsia and antepartum hemorrhage [8,9]. Increased concentrations of fetal fibronectin in cervical-vaginal secretions and shortness of cervical length are strong indicators of spontaneous PTB [10,11]. Prematurity is a significant contributor to prenatal death and a leading cause of newborn morbidities [12]. Though babies with premature birth can survive, premature babies have significant long-term health problems, including the potential effect on the fetus’s overall well-being, behavioral and cognitive characteristics, impaired neurodevelopmental, and significant gastrointestinal and respiratory diseases [1,13,14].

Globally, screening and prevention services are implemented in several countries intended to recognize high-risk women for PTB. For example, in Western Australia, ultrasonography is used to assess the risk of preterm birth based on cervical shortening and then to establish preventative measures for these complications. However, in East Africa, many preterm births continue to go unnoticed, and the underlying causes have not been identified in order to prevent their occurrence and associated complications, particularly in Somalia, where they are most prevalent.

In Somalia, the prevalence and risk factors of preterm births have not been well described. Thus, this study intends to investigate the maternal risk factors of preterm births in Mogadishu, Somalia, and, more importantly, identify target areas for future evidence-based interventions.

## 2. Materials and Methods

### 2.1. Study Design

The study employed an unmatched case-control study design adapted with modification [15]. The study was conducted in Mogadishu, Somalia, at four primary maternity hospitals: Benadir Hospital, S.O.S Hospital, Somali Sudanese Hospital, and Wardi community health center. All pregnant women admitted to the delivery ward of the four hospitals with gestational ages of 20 weeks or more with live singleton birth outcomes were included in this study. Prior to delivery, the data collection staff were present at every birth, and women were asked about their frequency of prenatal care visits and service utilization. Maternal medical and obstetrical variables were recorded with the assistance of the department’s physician and midwife.

### 2.2. Study Papulation

A total of 518 women gave birth during the study period. Mothers that delivered twin babies were omitted from the study, and they comprised a total of 13 twin neonates, so the sample size became 505 singleton neonates. In addition to the elimination of post-term neonates, the exact sample size taken for this study was 499. In this study, only live singletons were included: those deliveries with GA less than 37 weeks were considered as cases with a total sample of 70 neonates, and all term deliveries with a GA of 37 to 42 weeks were considered as controls, being the total of 429 neonates. The data was collected using a structured survey questionnaire. The questionnaire was divided into sections: maternal generation information, maternal characteristics related to the uptake and utilization of ANC services, and maternal medical and obstetric information.

### 2.3. Statistical Analysis

The data was entered into a Microsoft Office Excel database and exported to SPSS 26 version to analyze the data. The study employed cross-tabulation analysis to compare the proportion of each variable. In addition, binary logistic regression was used in order to find the relationship between the maternal factors associated with preterm birth and were presented as odds ratio (OR), 95% confidence interval (CI), and *p*-value. A *p*-value is considered statistically significant if its (<0.05).

### 2.4. Ethical Consideration

The study obtained ethical approval from the Jamhuriya University institutional ethics committee in 2020. Prior to the interviews, verbal comments were taken from mothers after they were fully informed about the objectives of the project while ensuring privacy and the confidentiality of participants.

## 3. Results

Maternal variables that influence preterm birth outcomes were studied among women attending four Mogadishu maternity hospitals between February and April of 2021. All these findings are concluded in the tables below.

### 3.1. General Characteristics

The majority of participants (*n* = 347/499, 69.5%) were between the ages of 21 and 39 years, representing 46 (9.2%) of the cases and 301 (60.3%) of the controls. In terms of marital status, 13.4% of cases and 84.8% of controls were married. Some 8.6% of cases and 40.3% of controls were of low socioeconomic status. Mothers’ education revealed that 12% of cases and 70.5% of controls had low literacy rates. Female genital mutilation (FGM), a type of female circumcision that involves the complete removal of the female genitalia for non-medical reasons, was performed in 12.8% of cases and 41.3% of controls. Close to 80% of pregnant women of multigravida status were 10.4% of cases and 69.1% of controls. However, the maternal parity numbers varied. For instance, 8.8% of cases and 57.1% of controls were multiparous, while 2.4% of cases and 15.8% of controls were primiparous, and 2.8% of cases and 13% of controls were nulliparous. The mean maternal age of cases was 25 years, while controls were 26 years. In terms of the child’s gender, male babies made up 7.4% of cases and 41.7% of controls, while female babies made up 6.6% of cases and 44.3% of controls. In terms of birth weight, 6.2% of cases and 4.8% of controls were under 2500 g, while 7.8% of cases and 81.2% of controls were over 2500 g. Table 1 summarizes the socio-demographic characteristics of preterm birth mothers.

### 3.2. Maternal Antenatal Care Characteristics and Their Association with Preterm Birth

A greater population of 241 (48.3%) pregnant women, which is nearly half of the participants, attended 1–2 ANC visits, representing 26 (5.2%) of the cases and 215 (43.1%) of the controls. We found that frequent ANC visits (>3 visits) were three times less likely to result in preterm birth (OR = 3.664, 95% CI: 1.723–7.791, *p*-value = 0.001). Thirty-seven (7.4%) of the cases and 301 (60.3%) of the controls received blood pressure (BP) measurements. The BP measurement at ANC was 2 times less likely to be premature delivery (OR = 2.097, 95% CI: 1.256–3.503, *p*-value = 0.005). Regarding other ANC-provided services such as tetanus toxoid vaccination, 37 (7.4%) of the cases and 293 (58.7%) of the control did receive vaccination and had nearly two times less chance of developing preterm delivery. Furthermore, half of all preterm cases (35/499, 7.0%) and more than two-quarters (315/499, 63.1%) of controls had received hemoglobin measurements at ANC and 2.7 less likely to get preterm birth (OR = 2.76, 95% CI: 1.651–4.625, *p*-value = 0.000). Thirty-nine (7.8%) of the cases and (133/499, 26.7%) of the controls received no iron and folic acid (IFA) supplementation during their antenatal care visits, while those who received iron and folic acid were at 2.8 times less risk of preterm birth (OR = 2.800, 95% CI: 1.674–4.682, *p*-value = 0.000). The mothers who started iron and folic acid in the last trimester were almost four times less likely to result in preterm birth (OR = 3.945, 95% CI: 1.942–8.016, *p*-value = 0.000). Another significant variable was the availability of ultrasound checks during antenatal care visits. Thirty-nine (7.8%) of the cases and 119 (23.8%) of the controls had no ultrasound checks during their current pregnancy compared to 31(6.2%) of cases and 310 (62.1%) of controls who did have an ultrasound check. Subsequently, those with ultrasound checks had a remarkably three times lower chance of becoming preterm cases than their counterparts (OR = 3.27, 95% CI: 1.955–5.495, *p*-value *=* 0.000). Table 2 summarizes maternal antenatal care characteristics.

### 3.3. Maternal Medical Characteristics with their Association of Preterm Birth

Maternal anemia was prevalent in this study. The majority of the mothers with anemia were controls, with 313 (62.7%) compared to 55 (11%) of the cases. According to the type of anemia, participants with mild anemia had 28 (5.6%) of the cases and 160 (32.1%) of the controls, while those with moderate anemia were 18 (3.6%) of the cases and 122 (24.4%) of the controls were diagnosed with moderate anemia, and 9 (1.8%) of the cases and 31 (6.2%) of the controls had severe anemia. Our study included medical illnesses such as hypertension and diabetes. For instance, hypertension was present in 0.8% of the cases and 2% of the controls, yet it was not significant for this study. However, a history of previous preterm birth was present in a total of 26.3%, with 5.2% of cases and 21% of controls, and had more likely to be premature birth (OR = 0.548, 95% CI: 0.322–0.934, *p*-value = 0.027). A history of preeclampsia was in 3% of cases and 4.8% of controls, which were more likely to result in preterm birth (OR = 0.217, 95% CI: 0.107–0.439, *p*-value = 0.000). Moreover, a history of miscarriage was found among 13(2.6%) of the cases and 67 (13.4%) of the controls. Table 3 summarizes maternal medical characteristics.

### 3.4. Maternal Obstetric Factors and Their Association with Preterm Births

Out of 57 deliveries (11.4%) that were partograph monitored, 19 babies (3.8%) were born prematurely, while 38 babies (7.6%) were born full term. Partograph-monitored labors were less likely to result in preterm birth (OR = 0.261, 95% CI: 0.140–0.487, *p*-value = 0.000). For FGM, 12.8% were cases while 41.3% were controls and more likely to risk to be premature delivery (OR = 0.87,95%CI = 0.37–0.24, *p*-value = 0.000). Regarding the way of presentation, the majority of the participants (13.2% of the cases and 79.6% of the control groups) were in cephalic presentation, while 0.8% of the cases and 5.8% of the controls were in breech presentation. About 12% of preterm cases and 67.5% of controls were vaginal delivery, while 2% and 18.4% were cesarean sections. According to the type of delivery, spontaneous vaginal delivery accounted for the majority, 64.5% of the controls and 11.2% of the cases, respectively, while 0.8% of cases and 3% of controls were assisted delivery, respectively. Those with cesarean section, especially with an emergency cesarean, were the majority, with 1.6% of cases and 10.6% of controls. Obstetric complications in this study were significantly associated with preterm birth, being a total of 19.2%, of which 4.4% were cases and 14.8% controls, which were more likely to lead to preterm birth (OR = 0.455, 95% CI: 0.259–0.799, *p*-value = 0.006) Regarding the type of obstetrical complication, preterm cases accounted for 1% of those with antepartum and 1% of those with postpartum hemorrhages compared to 2.6% and 4% of the controls, respectively. We recorded 0.8% and 2.6% of maternal outcomes as deaths. Table 4 illustrates the maternal obstetrical factors.

## 4. Discussion

Our study employed an unmatched case-control approach to assessing maternal risk factors for premature delivery in 499 live singletons born in Mogadishu, Somalia. Adequate prenatal care, FGM, partograph usage, prior preterm delivery and preeclampsia, obstetrical complications, and low birth weight neonates were all significantly related to preterm births.

First, the study examined the effect of prenatal care services on preterm births. Even though the majority of women received regular prenatal care, 27.5% of these women did not receive any prenatal care during their current pregnancies. Our findings suggest that an increased risk of premature birth was found to be associated with inadequate antenatal care. Mothers who had three or more ANC visits had a nearly four-fold lower risk of preterm birth and a higher chance of having a term baby.

There was a strong correlation between the amount of iron and folic acid pregnant women consumed and their risk of prematurity [16]. Premature births were found to be lower among women who began IFA in the third trimester compared to those who began IFA in the first trimester. This finding is in contrast to the findings of previous studies, which found that starting IFA during the first trimester had the best outcomes. According to Jiang et al., women with inadequate antenatal care had a 2.87-fold increased risk of preterm birth compared to women with regular prenatal care [7]. Identical discoveries were made in neighboring countries. In Ethiopia, pregnant women with a single ANC visit were found to have a higher risk of contracting PTB than those with four or more visits [15,17]. In Tanzania, women without prenatal care were nine times more likely to deliver premature babies than those with four ANC visits [9]. The study examined the influence of ultrasound monitoring during pregnancy. Although the majority of the women underwent ultrasound checks, some of them (31.7%) did not get ultrasound monitoring. There was a three-fold reduction in the chance of preterm birth for mothers who got an ultrasound during their first trimester. Ultrasound evaluation in early pregnancy during prenatal care will increase the likelihood of detecting pregnancy abnormalities and decrease the risk of premature birth. A Ugandan study showed that the use of ultrasonography in prenatal care can detect pregnancy problems and promote a safe birth [18]. In addition, other ANC services were also important for pregnancy outcomes. Pregnant women who had Bp measurement, TT vaccination, and urine analysis for maternal infection strongly favored full-term births. In conclusion, inadequate antenatal care during pregnancy increases the risk of PTB and that the absence of adequate ANC services decreases the likelihood of detecting preterm birth risk and delivering appropriate preventive interventions [19].

Female genital mutilation (FGM) is a problem that affects nearly half of pregnant women in this study. Findings from our study show that women who have had FGM are more likely to give birth prematurely, an association that has not been identified in other studies. Complications of FGM are most likely to blame, such as extensive scarring and narrowing of the birth canal, along with ongoing colonization by germs that can cause infections of the bladder and other diseases [20,21]. More research is needed to determine the precise link between FGM and premature birth.

Pregnant women with obstetric complications and preeclampsia had a higher preterm birth risk. Of the 7% of the women with preeclampsia in our study, 3% of their births ended prematurely. Prior research from Yemen and Tanzania supported our findings that preeclampsic mothers had a four-fold and seven-fold increased risk of premature delivery, respectively [22,23]. Another Ugandan study found that women with preeclampsia were 16 times more likely to deliver prematurely than normotensive women [24]. Pregnant women having a history of preterm births were more likely to do so again. We found that 26.3% of mothers having a history of preterm births had 5.2% of preterm babies during the current pregnancy. Previous research conducted in Korea, Italy, and Thailand found that having a prior history of preterm birth increases the likelihood of giving another premature birth by three to four folds [25]. Another study from Ethiopia and Iran also correlated prior history of preterm birth and prematurity [17,19,20,26]. Despite the fact that the likelihood that women who have previously given birth prematurely would do so again is growing, the underlying mechanism is unclear and must be thoroughly addressed. However, ultrasound monitoring and adequate prenatal care visits could help identify the risk and prevent it.

The study’s strength was that it was conducted at four primary maternity hospitals. Two of the hospitals (Benadir and S.O.S) are considered referral hospitals for high-risk pregnancies and women with complications. In addition, to our knowledge, this is the first study from the study area which investigated the maternal risk factors for preterm birth in Mogadishu, Somalia. One of the study’s drawbacks is that it was carried out in a hospital environment, where hospital delivery rates are low and might not represent the general population. Future studies should address the link between FGM and prematurity, which is critical.

## 5. Conclusions

This study showed that there is a high prevalence of prematurity. Understanding and recognizing maternal risk factors associated with preterm birth may aid in the prevention of preterm birth complications. This study emphasizes the importance of prenatal care, obstetric complications, and FGM on preterm birth. To prevent the occurrence and associated long-term complications of prematurity, the Ministry of Health should engage in community education to increase ANC service utilization, as well as improve health institutions’ capacity to identify and treat obstetric complications.

## Figures and Tables

**Table 1:** Socio-demographic characteristics of pregnant women in Mogadishu (*N* = 499).

Variable	Total (%)	Case (%)	Control (%)
**Hospital name**			
Wardi Community Hospital	19 (3.8)	0 (0)	19 (3.8)
Banadir Hospital	361 (72.3)	50 (10.0)	311 (62.3)
S.O.S Mother and Child Hospital	63 (12.6)	15 (3.0)	48 (9.6)
Somali Sudanese Specialist Hospital	56 (11.2)	5 (1.0)	51 (10.2)
**Maternal Age Group**			
<20 years	137 (27.5)	23 (4.6)	114 (22.8)
21–39 years	347 (69.5)	46 (9.2)	301 (60.3)
>40 years	15 (3)	1 (0.2)	14 (2.8)
**Residence**			
Urban	473 (94.8)	66 (13.2)	407 (81.6)
Rural	17 (3.4)	3 (0.6)	14 (2.8)
Internally displaced populations (IDPs)	9 (1.8)	1 (0.2)	8 (1.6)
**Maternal Gravida**			
Primigravida	102 (20.4)	18 (3.6)	84 (16.8)
Multigravida	397 (79.6)	52 (10.4)	345 (69.1)
**Maternal employed**			
No	449 (90)	63 (12.6)	386 (77.4)
Yes	50 (10)	7 (1.4)	43 (8.6)
**Parity group**			
Nulliparous	79 (15.8)	14 (2.8)	65 (13.0)
Primiparous	91 (18.2)	12 (2.4)	79 (15.8)
Multiparous	329 (65.9)	44 (8.8)	285 (57.1)
**Mother’s Female genital mutilation (FGM)status**			
No	229 (45.9)	6 (1.2)	223 (44.7)
Yes	270 (54.1)	64 (12.8)	206 (41.3)
**Marital Status**			
Married	490 (98.2)	67 (13.4)	423 (84.8)
Divorced	7 (1.4)	2 (0.4)	5 (1.0)
Windowed	2 (0.4)	1 (0.2)	1 (0.2)
**Women Education**			
Illiterate	412 (82.6)	60 (12.0)	352 (70.5)
School	82 (16.4)	9 (1.8)	73 (14.6)
University	5 (1.0)	1 (0.2)	4 (0.8)
**Mather Socioeconomic Status**			
High	36 (7.2)	3 (0.6)	33 (6.6)
Middle	219 (43.9)	24 (4.8)	195 (39.1)
Low	244 (48.9)	43 (8.6)	201 (40.3)
**Mother’s body mass index (BMI)**			
Normal	412 (82.6)	58 (11.6)	354 (69.1)
Under Weight	35 (7.0)	5 (1.0)	30 (6.0)
Over Weight	52 (10.4)	7 (1.4)	45 (9.0)
**Sex of child**			
Male	245 (49.1)	37(7.4)	208 (41.7)
Female	254 (50.9)	33(6.6)	221 (44.3)
**Birth weight groups**			
<2500 grams	55 (11)	31 (6.2)	24 (4.8)
>2500 grams	444 (89)	39 (7.8)	405 (81.2)

**Table 2:** Maternal Antenatal care utilization during current pregnancy among women attending four maternal hospitals in Mogadishu, Somalia.

Maternal ANC Characteristics	Total (%)	Case and Control		OR	95% CI	*p*-Value
		Case (Preterm)	Control (Term)			
		*N* (%)	*N* (%)			
**Maternal age groups**						
<20	137 (27.5)	23 (4.6)	114 (22.8)	**Reference**	**Reference**	**Reference**
21–39	347 (69.5)	46 (9.2)	301 (60.3)	1.320	0.766–2.277	0.318
>40	15 (3.0)	1 (0.2)	14 (2.8)	2.825	0.354–22.556	0.327
**Frequency of ANC visits**						
No Visits	137 (27.5)	34 (6.8) 26 (5.2)	103 (20.6)	Reference	Reference	Reference
1–2	241 (48.3)		215 (43.1)	2.730	1.556–4.789	0.000
3–4	121 (24.2)	10 (2.0)	111 (22.2)	3.664	1.723–7.791	0.001
**Maternal urine analysis at ANC**						
No	206 (41.3)	39 (7.8)	167 (33.5)	Reference	Reference	Reference
Yes	293 (58.7)	31 (6.2)	262 (52.5)	1.974	1.185–3.287	0.009
**Tetanus Toxoid (TT) vaccination status**						
No	169 (33.9)	33 (6.6)	136 (27.3)	Reference	Reference	Reference
Yes	330 (66.1)	37 (7.4)	293 (58.7)	1.922	1.152–3.205	0.012
**Hemoglobin measurement ANC**						
No	149 (29.9)	35 (7.0)	114 (22.8)	Reference	Reference	Reference
Yes	350 (70.1)	35 (7.0)	315 (63.1)	2.763	1.651–4.625	0.000
**Ultrasound check during current pregnancy**						
No	158 (31.7)	39 (7.8)	119 (23.8)	Reference	Reference	Reference
Yes	341 (68.3)	31 (6.2)	310 (62.1)	3.277	1.955–5.495	0.000
**Iron + folic acid supplement intake**						
No	172 (34.5)	39 (7.8)	133 (26.7)	Reference	Reference	Reference
Yes	327 (65.5)	31 (6.2)	296 (59.3)	2.800	1.674–4.682	0.000
**When did you started iron + folic supplement**						
Non	172 (34.5)	39 (7.8)	133 (26.7)	Reference	Reference	Reference
First trimester	46 (9.2)	6 (1.2)	40 (8.0)	1.955	0.772–4.951	0.157
Second trimester	122 (24.4)	14(2.8)	108 (21.6)	2.262	1.168–4.382	0.016
Third trimester	159 (31.9)	11 (2.2)	148 (29.7)	3.945	1.942–8.016	0.000
**Blood pressure measurement at ANC**						
No	161 (32.3)	33 (6.6)	128 (25.7)	Reference	Reference	Reference
Yes	338 (67.7)	37 (7.4)	301 (60.3)	2.097	1.256–3.503	0.005

OR = Odds ratio, CI = Confidence interval, ANC = Antenatal care.

**Table 3:** Maternal medical factors and preterm birth among pregnant women delivered in four hospitals, Mogadishu, Somalia.

Maternal Medical Characteristics	Total (%)	Case And Control		OR	95% CI	*p*-Value
		Case (Preterm)	Control (Term)			
		*N*%	*N*%			
**Presence of Anemia**						
No	108 (21.6)	15 (3.0)	93 (18.6)	**Reference**	**Reference**	**Reference**
Yes	368 (73.7)	55 (11.0)	313 (62.7)	0.918	0.496–1.700	0.785
**Anemia Type**						
No Anemia	108 (21.6)	15 (3.0)	93 (18.6)	**Reference**	**Reference**	**Reference**
Mild (9.0–10.9) g/dl	188 (37.7)	28 (5.6)	160 (32.1)	0.922	0.468–1.814	0.813
Moderate (7.0–8.9) g/dl	140 (28.1)	18 (3.6)	122 (24.4)	1.093	0.523–2.283	0.813
Severe (<7) g/dl	40 (8.0)	9 (1.8)	31 (6.2)	0.556	0.221–1.395	O.211
**Medical conditions**						
None	482 (96.6)	66 (13.2)	416 (83.4)	**Reference**	**Reference**	**Reference**
Hypertension	14 (2.8)	4 (0.8)	10 (2.0)	0.397	0.121–1.301	0.127
Diabetes mellitus	3 (0.6)	0 (0.0)	3 (0.6)	256301297.2	0.000	0.999
**History of previous preterm birth**						
No	368 (73.7)	44 (8.8)	324 (64.9)	**Reference**	**Reference**	**Reference**
Yes	131 (26.3)	26 (5.2)	105 (21.0)	0.548	0.322–0.934	0.027
**History of preeclampsia**						
No	460 (92.2)	55 (11.0)	405 (81.2)	**Reference**	**Reference**	**Reference**
Yes	39 (7.8)	15 (3.0)	24 (4.8)	0.217	0.107–0.439	0.000
**History of miscarriage**						
No	419 (84.0)	57 (11.4)	362 (72.5)	**Reference**	**Reference**	**Reference**
Yes	80 (16.0)	13 (2.6)	67 (13.4)	0.812	0.421–1.564	0.533
**Maternal infections**						
No	448 (89.8)	65 (13.0)	383 (76.8)	**Reference**	**Reference**	**Reference**
Yes	51 (10.2)	5 (1.0)	46 (9.2)	1.561	0.598–4.076	0.363

OR = Odds ratio, CI = Confidence interval, g/dl = grams per deciliter.

**Table 4:** Maternal obstetric factors and preterm birth among pregnant women delivered in the four hospitals, Mogadishu, Somalia.

Obstetric Features with Outcome	Total (%)	Case And Control		OR	95% CI	*p*-Value
		Case (Preterm)	Control (Term)			
		*N* (%)	*N* (%)			
**Use Partograph**						
No	462 (88.6)	51 (10.2)	391 (78.4)	Reference	Reference	Reference
Yes	57 (11.4)	19 (3.8)	38 (7.6)	0.261	0.140–0.487	0.000
**FGM status**						
No	229 (45.9)	6 (1.2)	223 (44.7)	Reference	Reference	Reference
Yes	270 (54.1)	64 (12.8)	206 (41.3)	0.87	0.37–0.204	0.000
**Way Of Presentation**						
Cephalic	463 (92.8)	66 (13.2)	397 (79.6)	Reference	Reference	Reference
Breach	33 (6.6)	4 (0.8)	29 (5.8)	1.205	0.410–3.540	0.734
Other	3 (0.6)	0 (0.0)	3 (0.6)	268567606.1	0.000	O.999
**Occurrence of obstetric Complications**						
No	403 (80.8)	48 (9.6)	355 (71.1)	Reference	Reference	Reference
Yes	96 (19.2)	22 (4.4)	74 (14.8)	0.455	0.259–0.799	0.006
**Types Of Obstetric Complications**						
None	403 (80.8)	48 (9.6)	355 (71.1)	Reference	Reference	Reference
APH	18 (3.6)	5 (1.0)	13 (2.6)	0.352	0.120–1.030	0.057
PPH	25 (5.0)	5 (1.0)	20 (4.0)	0.541	0.194–1.508	0.240
PROM	3 (0.6)	3 (0.6)	0 (0.0)	0.000	0.000	0.999
Obstructed labor	22 (4.4)	2 (0.4)	20 (4.0)	1.352	0.306–5.966	0.690
Others	28 (5.6)	7 (1.4)	21 (4.2)	0.406	0.164–1.005	0.051
**Maternal Outcome**						
Alive	482 (96.6)	66 (13.2)	416 (83.4)	Reference	Reference	Reference
Death	17 (3.4)	4 (0.8)	13 (2.6)	0.516	0.163–1.629	0.259
**Types Of Delivery**						
Spontaneous	378 (75.8)	56 (11.2)	322 (64.5)	Reference	Reference	Reference
Assisted	19 (3.8)	4 (0.8)	15 (3.0)	0.652	0.209–2.037	0.462
Elective C/S	41 (8.2)	2 (0.4)	39 (7.8)	3.391	0.796–14.444	0.099
Emergency C/S	61 (12.2)	8 (1.6)	53 (10.6)	1.152	0.520–2.553	0.727
**Mode Of Delivery**						
Vaginal	397 (79.6)	60 (12.0)	337 (67.5)	Reference	Reference	Reference
C/S	102 (20.4)	10 (2.0)	92 (18.4)	1.638	0.807–3.325	0.172

OR = Odds ratio, CI = Confidence interval, FMG = Female genital mutilation, C/S = Cesarean section, APH = Antepartum haemorrhage, PPH = Postpartum hemorrhage, PROM = Premature rupture of membranes.

## Data Availability

All relevant data are within the paper and its Supporting Information files.
